# Effect of ultrasound training of physicians working in the prehospital setting

**DOI:** 10.1186/s13049-016-0289-1

**Published:** 2016-08-04

**Authors:** Charlotte Loumann Krogh, Jacob Steinmetz, Søren Steemann Rudolph, Rasmus Hesselfeldt, Freddy K. Lippert, Peter A. Berlac, Lars S. Rasmussen

**Affiliations:** 1 Department of Anaesthesia, Centre of Head and Orthopaedics, Rigshospitalet, University of Copenhagen, Blegdamsvej 9, 2100 København Ø, Denmark; 2 Emergency Medical Services Copenhagen, The Capital Region of Denmark, Telegrafvej 5, 2750 Ballerup, Denmark; 3 Sankt Thomas Allé 13, 3 th., 1824 Frederiksberg C, Denmark

**Keywords:** Prehospital care, Ultrasound, Emergency treatment, Training, Assessment, E-learning

## Abstract

**Background:**

Advances in technology have made ultrasound (US) devices smaller and portable, hence accessible for prehospital care providers. This study aims to evaluate the effect of a four-hour, hands-on US training course for physicians working in the prehospital setting. The primary outcome measure was US performance assessed by the total score in a modified version of the Objective Structured Assessment of Ultrasound Skills scale (mOSAUS).

**Methods:**

Prehospital physicians participated in a four-hour US course consisting of both hands-on training and e-learning including a pre- and a post-learning test. Prior to the hands-on training a pre-training test was applied comprising of five videos in which the participants should identify pathology and a five-minute US examination of a healthy volunteer portraying to be a shocked patient after a blunt torso trauma. Following the pre-training test, the participants received a four-hour, hands-on US training course which was concluded with a post-training test. The US examinations and screen output from the US equipment were recorded for subsequent assessment. Two blinded raters assessed the videos using the mOSAUS.

**Results:**

Forty participants completed the study. A significant improvement was identified in e-learning performance and US performance, (37.5 (SD: 10.0)) vs. (51.3 (SD: 5.9) *p* = < 0.0001), total US performance score (15.3 (IQR: 12.0-17.5) vs. 17.5 (IQR: 14.5-21.0), *p* = < 0.0001) and in each of the five assessment elements of the mOSAUS.

**Conclusion:**

In the prehospital physicians assessed, we found significant improvements in the ability to perform US examinations after completing a four-hour, hands-on US training course.

## Background

Today US is considered an integral diagnostic adjunct in modern emergency medicine and trauma care [1]. While in-hospital healthcare providers have been using US for decades for detecting pneumothorax and haemoperitoneum, the prehospital system has only recently adopted this modality [2–7]. Advances in technology have reduced cost and size of US devices making them available for prehospital care providers.

Early diagnosis in the prehospital setting is essential in order to guide triage and choice of transfer modality [8]. US examination has shown to be both feasible and accurate in the prehospital setting although several environmental barriers such as noise and limited workspace have to be overcome [9, 10].

US examinations are highly operator dependent, yet prehospital US examinations are mostly performed by non-radiologists with variable US experience. To overcome this dilemma point-of-care US protocols are characterized by addressing specific (often dichotomous) questions [11]. This approach makes point-of-care US suitable for the prehospital setting enabling clinical decision making without delay in patient treatment. However, the minimum training to achieve sufficient competence may vary considerably between individual physicians. Hence the number of examinations required before independent practice is properly an insufficient measure of competence [12].

Various types of educational programs have been developed, mainly focusing on didactic training and hands-on experience [13, 14]. In order to obtain sufficient competence in US a validated measure of competence must be used in assessment of skills.

The aim of this study was to evaluate the effect of a four-hour, hands-on US training course in combination with e-learning. US performance was assessed using a modified version of a newly developed scale, the Objective Structured Assessment of Ultrasound Skills (OSAUS) scale for US performance [15, 16].

## Methods

### Study design and context

This was a prospective study investigating the effect of an US course for prehospital physicians affiliated to the Copenhagen Emergency Medical Services’ (EMS) five Mobile Emergency Care Units (MECU). The study was conducted in relation to the implementation of US at the MECU. The participants’ US skills were tested before and after receiving a four-hour, hands-on US training course. The primary outcome measure was US performance assessed by the total score of the mOSAUS scale.

### Sample and setting

Sixty-one physicians from the MECU were invited to participate in the study. The MECU is an advanced life support (ALS) unit staffed with a consultant anaesthesiologist and paramedic as an assistant. The physicians were all experienced consultant anaesthesiologists. The MECU services the entire Capital Region, covering 2.561 km^2^ and comprises of a mixed urban and rural population of 1.7 million. The five MECUs have an annual mission rate of approximately 19.000 dispatches, of which nearly 3.000 are trauma related. To accommodate work schedules, the physicians were asked to sign up for one of seven possible courses in order to attend the hands-on training.

### E-learning

Prior to the hands-on training, the participants were provided with a registration code and a license key to access an online e-learning program containing seven interactive modules: Introduction, Basic Ultrasound, Equipment and Skills, Lung Ultrasound, Focused Assessment with Sonography for Trauma, Focus Accessed Transthoracic Echocardiography and Peripheral Ultrasound Guided Vascular Access. The participants could complete the e-learning program before the hands-on training when convenient. Each module had a duration of 10–20 min and the total completion time was approximately 110 min. The e-learning program covered US theory and techniques in combination with photos, video clips, and tests to ensure retention of knowledge. To access the e-learning program the participants first had to complete a pre-learning test, followed by an identical post-learning test. This test consisted of 56 questions in basic and advanced US theory presented as true or false statements, pairing phrases together and matching letters to structures on US images. The pre-learning test could only be taken once, while the post-learning test could be repeated.

### Pre-training test

Initially, the participants completed a questionnaire regarding their level of US experience. Each participant was then presented with five short US clips containing the following pathological conditions: free fluid in Morison's pouch, left ventricular hypokinesis, absent lung sliding, right ventricular dilatation and left ventricular hypokinesis (alternate case). The participant was informed about the specific projection of the clip and was then requested to detect if pathology was present, and if so which specific pathology. Finally, each participant completed a five-minute examination of a healthy volunteer portraying to be a shocked patient after blunt torso trauma. The pre-training test US examination was obtained using a portable US device (GE LOGIQ S7 with a Convex Probe 1.8–5.5 MHz, Sector probe 1.6–5.5 MHz and Linear 4.2–12 MHz). The participant was given a short introduction to the US equipment including changing transducers before commencing the examination. The examinations and screen output from the US equipment were recorded for subsequent assessment. The participants were not given any guidance during the test scenario nor feed-back on their performance afterwards. For the purpose of blinding of the raters the participants’ faces were not filmed.

### Hands-on training

The hands-on training course began with a brief introduction followed by a 30-min workshop covering US concepts and principles for scanning in the pre-hospital setting. The hands-on training was conducted by four emergency US instructors in small groups establishing a participant-to-instructor ratio of 4: 1. Each group rotated through four instructor-led stations with healthy volunteers demonstrating 12 simulated case scenarios. The contents and pathological findings of the 12 cases were: Abdominal pain/ abdominal aortic aneurysm, chest trauma / pneumothorax, chest pain / pulmonary embolism, dyspnea / pulmonary edema, cardiac arrest / pericardial tamponade, abdominal trauma / free fluid in Morison’s pouch, syncope / pericardial hematoma, dyspnea / pleural effusion, torso trauma / pneumothorax, free fluid in Morison’s pouch, perisplenic and pelvic, dyspnea / pleural effusion and pulmonary embolism, chest pain / myocardial infarction and chest pain / pericardial and pleural effusion. For each case scenario the participants were given a short case presentation and were encouraged to perform a focused US examinations according to the clinical situation. Non-pathological findings corresponded to findings in the volunteer whereas pathology was displayed by the instructor on an iPad. During the first 30 min of the hands-on training the participants performed US examinations using the same US device as in the pre-training test (GE LOGIQ S7), whereas the following two-hour training session was done using a portable, pocket-sized US device (GE Vscan with Dual Probe). This device was used for US examination in the remaining part of the hands-on training. Continuous feedback and tips for image optimization were provided by the instructors throughout the hands-on training.

### Post-training test

After completion of hands-on training, the pre-training test was re-administered. The post-training test was identical in both content and structure of the pre-training test and was conducted in the same manner. As with the pre-training test, the post-training test performance was recorded for subsequent assessment.

### Video processing

Video recordings of the examinations (Sony Exmor Handycam video recorder) and the screen outputs (MediCapTM USB200 medical recorder) from the US machine were merged using Final Cut Pro 7 video-editing software to one sound muted anonymized video with the mOSAUS rating scale presented as a tick-box. This allowed both the technical performance and the US image to be reviewed simultaneously. All video clips were assigned a randomized number (created using https://www.random.org/) and uploaded into a password-protected web page from which the raters could access them.

### Assessment of performance

**Fig. 1:**
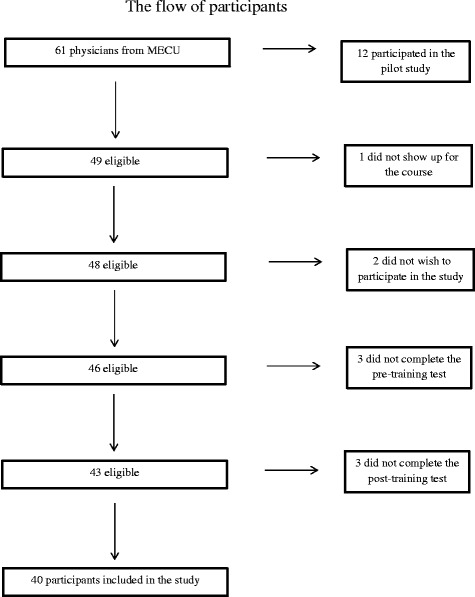
Flow diagram demonstrating the number of eligible participants included in the study

Two independent blinded raters (SSR and RH) with experience in US including emergency US assessed the US performances using the mOSAUS scale. The OSAUS scale originally consisted of 7 elements. We modified the scale assessing: Applied knowledge of US equipment, image optimization, systematic examination, interpretation of images and identification of at least one pathological finding in each of five ultrasound videos. Prior to the assessment, the two raters participated in a 90-min training session, where they received a short introduction to the OSAUS rating scale. Subsequently, they independently assessed six pilot videos and discussed their ratings until consensus was reached. The pilot videos were randomly selected and enlisted for the pilot study. The case and examinations in the pilot videos were identical to the main study. However, these six participants were not included in the study sample (Fig. 1). The two raters were provided with access to the video clips via e-mail. The video clips were provided in random order using the participants’ ID number. The raters were blinded to the participants’ identity, the sound recordings, whether the recorded session was a pre-training test or a post-training test, and the rating given by the other expert. Subsequently, data were transferred from the web page to the database.

### Data analyses

Baseline characteristics were reported as proportions or median with interquartile range and compared with Chi-square test or Mann-Whitney Test.

Pre-training performance and post-training performance were compared using paired t-tests and changes are reported as mean difference with 95 % confidence interval.

Interrater reliability was examined using the intraclass-correlation coefficient (ICC). Data were analyzed using SAS statistical software, version 9.2 (SAS Institute, Cary, NC) and graphpad.com.

A previous study using the OSAUS rating method found that novices (in sonography) performing four cases (maximum score of 100 points) had a mean score (SD) of 17 (8.4) [16]. Physicians with intermediate experience (in sonography) had a score of 30 (SD 10.1). Hence, we estimated that novices’ performance in one case instead of four would be 4.3 (2.5), and intermediate experienced persons’ score would be 7.5 (2.5). In this paired study, we aimed to detect a change in performance and we calculated that a sample of approximately 20 persons would be needed to detect a change of 3 points in the total score in the mOSAUS scale with a power of at least 80 % at the 5 % significance level, assuming that the SD for change was around 3 points as well. *P*-values < 0.05 were considered statistically significant.

## Results

The entire hands-on training program was completed by 40/61 (66.7 %) participants. Of these, 27/40 (67.5 %) completed the pre- and post-learning test of the e-learning.

The reasons for drop-outs in the hands-on training were mainly logistical reasons such as late arrival to the pre-training test and work related obligations immediately after the course (Fig. 1).

The participants and drop-outs had a broad range of background characters including differences in US experience prior to the course (Table 1).

**Table 1:** Demographics of participants and drop outs presented by number or mean followed by range or percentage in brackets

Variable	Participants	Drop-outs	P
	*N* = 40	*N* = 18	
Gender – males	29 (72.5)	15 (71.4)	0.93
Years of medical experience	17.5 (14–24.5)	20.5 (15–27)	0.56
	Missing data: *N* = 0	Missing data: *N* = 3	
Most common examination			0.87
Vascular access	13	6	
Nerve block	13	6	
Lung US	2	1	
EFAST	3	1	
FAST	3	0	
Cardiac US	1	0	
	Missing data: *N* = 5	Missing data: *N* = 4	
Prior US certification	16 (40.0)	11 (61.1)	0.14
	Missing data: *N* = 0	Missing data: *N* = 1	
Prior US courses			0.30
No	24	7	
1–2	12	9	
>2	4	2	
	Missing data: *N* = 0	Missing data: *N* = 0	
US examinations prior to the course			0.26
0–25	28	15	
26–99	7	1	
>99	3	1	
	Missing data: *N* = 2	Missing data: *N* = 1	

Comparing pre- and post-learning test results of the e-learning the participants obtained significantly higher post-test mean scores 37.5 (SD: 10.0) vs. 51.3 (SD: 5.9), *p* < 0.0001.

The participants demonstrated significant improvement in performance in both total score (17.5 [14.5–21.0] vs. 15.3 [12.0–17.5], *p* < 0.0001) and in each assessment element of the mOSAUS scale. The largest improvement was the categories “systematic examination” and “identification of at least one pathological finding” in the US videos (Table 2).

**Table 2:** Pre – and post-training test scores in each of the OSAUS assessment elements including total score and mean change 95 % CI

Category	Pre-training test median and (IQR)	Post-training test median and (IQR)	Mean change with 95 % CI	*P*-value
(1) Applied knowledge of ultrasound equipment	4.0 (3.0–4.0)	4.0 (3.5–5.0)	0.51 (0.25–0.77)	0.0003
(2) Image optimization	2.8 (2.5–3.5)	3.3 (2.5–4.0)	0.43 (0.16–0.69)	0.0024
(3) Systematic examination	3.5 (2.75–4.0)	4.0 (3.5–5.0)	0.69 (0.30–1.08)	0.001
(4) Interpretation of images	2.5 (2.0–3.5)	3.5 (2.5–4.0)	0.55 (0.21–0.89)	0.0021
(5) Identification of at least one pathological finding.	2.0 (2.0–2.5)	3.0 (2.25–3.0)	0.61 (0.40–0.82)	<0.0001
Total score	15.3 (12.0–17.5)	17.5 (14.5–21.0)	2.79 (1.76–3.81)	<0.0001

The interrater reliability concerning the total pre-training test score was good (ICC = 0.74). Reliability was highest in “identification of pathology” (ICC 0.90), “interpretation of images” (ICC 0.68) and “systematic examination” (ICC = 0.68), and somewhat lower concerning “image optimization” (ICC = 0.42) and “applied knowledge of US equipment” (ICC = 0.60).

## Discussion

This study demonstrates that a four-hour, hands-on US training course, preceded by e-learning, had a substantial impact on prehospital physicians’ US skills. Using two blinded expert raters with good interrater reliability we found significant improvements in performances in both e-learning, total score and in each assessment element of the mOSAUS scale. The greatest improvements in scores were in aspects related to systematic examination and identification of pathology.

A significant limitation of this study is the lack of a control group without training. The US course was a mandatory course for all MECU physicians making a randomized controlled trial not feasible. A substantial number of participants (43 %) were not included from the study sample due to failure to complete the study. This could have led to a selection bias. However, comparing baseline characters including *p*-values of the participants and the drop-outs we found the two groups to be comparable. The post-learning test of the e-learning could be repeated if desired, which could have artificially inflated the participants’ performance. Only 3/40 participants repeated the post-learning test and therefore less likely to affect our results considerably. Finally, the use of the same pre-training and post-training test in assessment of US skills may also be a limitation to this study. However, the participants were not given any guidance during the test situations nor feed-back on their test performance which reduce (but does not eliminate) learning acquired during the test session. For future assessments the study sample could be randomised in to one of two pre- and post- training tests which may remove the risk of confounding by using identical setup for pre- and post-training assessment.

The participants had various experience with the use of US. However, only one participant used the extended focused assessment with sonography for trauma (EFAST) examination regularly, which makes the participants comparable in regards to US skills related to the US examination. Finally, the performance of the participants was tested using an US device which was different from the portable US device used during the majority of the training course. However, in research of motor skill learning, one method to assess learning outcome is to test the newly achieved skills in another context (transfer of learning) in order to demonstrate sustainable skills [17]. Hence by modulating the context of the motor skills we were able to test the adaptive aspects related to the obtained skills during the training.

We found the largest improvement in scores in the categories systematic examination and identification of pathological findings in the US videos. These skills are specifically useful in the pre-hospital environment, as procedures performed on-scene should be obtained systematically with minimal delay of patients’ clinical course aiming to detect specific pathological findings.

During the course, the participants trained US skills on healthy volunteers, which may explain why less improvement was found for image optimization. On top of that, the participants were only allowed five minutes to perform the US examination, which may have influenced their prioritization of the time available. When assessing performance of US in experienced non-radiologists, image optimization deemed challenging [18]. Our study sample included mainly novices in emergency US examination, and important diagnostic information may be lost if image optimization is not done properly. Hence US training courses should emphasize the importance of image optimization before interpretation and clinical decision-making based on US examination.

Research on prehospital US is still at an early stage, but an increased focus on its importance and potential impact is appearing [19–21]. A consensus report from Fevang et al. defined the role of prehospital US as one of top five research priorities in physician-provided pre-hospital critical care. A key question to be addressed is *“How should providers achieve and maintain specific ultrasound skills?”* [20]. Several research groups have investigated educational programs in order to establish competences in prehospital US. The length of training ranged from two hour sessions to longitudinal two months training programs including didactic teaching, experiential training and advanced interactive online learning. [5, 13, 14, 22]. Our results are in accordance to other studies demonstrating improvement in US skills and image recognition post training.

A previous study exploring the reliability and validity of the OSAUS scale for point- of-care ultrasound performance found a high correlation between the physicians’ OSAUS score and the number of correct diagnoses demonstrated by a Spearman ρ of 0.76 (*P* < 0.001) [16]. This indicate that the OSAUS score is a solid measure of competence and may be capable of predicting diagnostic accuracy. However, we are unaware whether the improvement in this setting translates into significant clinical improvements, or if further training is needed to ensure this. Moreover, the individual physician’s competences in being able to perform independent practice is not proven by this study. We have demonstrated a statistically significant change in the OSAUS score after a brief training session, but future studies should aim to evaluate whether this measurable change is clinically significant.

Strategies to prolong retention and investigation of transfer of skills to the prehospital setting are desirable. Lind et al. investigated the hypothesis that e-learning could be used as a booster to maintain competences following an advanced life support (ALS) course, but found no significant effect of this intervention. The lack of social interaction was identified as the major cause predicting the use of e-learning [23]. Future studies that combine refresher courses and/or tests with e-learning may demonstrate prolonged retention of skills obtained during hands-on training.

In terms of application of knowledge, a recent study from Todsen et al. has demonstrated that learning outcome obtained during a four-hour, hands-on training course in abdominal ultrasound could transfer to diagnostic performance in patients. The results also indicated that more training was needed in order to reach sufficient levels of competence [24]. Further studies are required to investigate the application of US in the prehospital environment and the potential clinical benefit of prehospital US in patient management and outcome.

## Conclusion

In conclusion, we found significant improvements in the ability to perform US examination among physicians working in the prehospital setting after completing a four-hour, hands-on training course. Further studies are required to investigate the potential clinical benefit of US training for prehospital care providers and of its’ application in the clinical environment.

## Abbreviations

ALS, advanced life support; EFAST, extended focused assessment with sonography for trauma; EMS, emergency medical services; FAST, focused assessment with sonography for trauma; ICC, intraclass-correlation coefficient; MECU, mobile emergency care units; mOSAUS, modified objective structured assessment of ultrasound skills; OSAUS, objective structured assessment of ultrasound skills; SD, standard deviation; US, ultrasound
